# The Prevalence of Feline Hip Dysplasia, Patellar Luxation and Lumbosacral Transitional Vertebrae in Pedigree Cats in The Czech Republic

**DOI:** 10.3390/ani11092482

**Published:** 2021-08-24

**Authors:** Petra Černá, Joep Timmermans, Dominik Komenda, Ivana Nývltová, Pavel Proks

**Affiliations:** 1 Small Animal Clinic, Faculty of Veterinary Medicine, University of Veterinary Sciences, 612 42 Brno, Czech Republic; komendad@vfu.cz (D.K.); nyvltovai@vfu.cz (I.N.); proksp@vfu.cz (P.P.); 2 Department of Clinical Sciences, Colorado State University, Fort Collins, CO 80528, USA; 3 People’s Dispensary for Sick Animals, Stoneygate Ln, Gateshead NE10 0LX, UK; joepjelletimmermans@gmail.com

**Keywords:** HD, LTV, lameness, Maine Coon cats, congenital, orthopedic

## Abstract

**Simple Summary:**

Hip dysplasia, patellar luxation and lumbosacral transitional vertebra are not well described in cats, most likely because cats can often much better compensate for pelvic limb lameness and hide their pain and, as a result, owners are less likely to notice this condition. Pedigree cats at least 10 months old were recruited prospectively in this study to find the prevalence of feline hip dysplasia, patellar luxation and lumbosacral transitional vertebra. The prevalence of hip joint dysplasia in all pedigree cats was 46.7%, of which 78% of cats had bilateral dysplasia. Dysplasia was mainly from mild (grade 1) to moderate (grade 2); however, 6.1% of hip joints showed signs of severe hip dysplasia (grade 3) in Maine Coon and Siberian cats. Patellar luxation was noted in 32.7% of the pedigree cats, was present bilaterally in 91.4% and was grade 1 or 2 in most cats. The presence of lumbosacral transitional vertebra was noted in 7.5% of the pedigree cats. The high prevalence of hip dysplasia in pedigree cats should be considered and screening pedigree cats for hip dysplasia is recommended before they are used in breeding programs.

**Abstract:**

(1) Background: The aim of this study was to find the prevalence of feline hip dysplasia (HD), patellar luxation and lumbosacral transitional vertebra (LTV) in pedigree cats in the Czech Republic. (2) Methods: 107 pedigree cats at least 10 months old were recruited prospectively at the Small Animal Clinic at the University of Veterinary and Pharmaceutical Sciences Brno, CZ, between April 2019 and July 2020. (3) Results: The prevalence of hip joint dysplasia in all pedigree cats was 46.7%, of which 78% of cats had bilateral dysplasia. The HD was mainly from mild (grade 1) to moderate (grade 2); however, 6.1% of hip joints showed signs of severe HD (grade 3) in Maine Coon and Siberian cats. Patellar luxation was noted in 32.7% of the pedigree cats, was present bilaterally in 91.4% and was grade 1 or 2 in most cats. The presence of LTV was noted in 7.5% of pedigree cats. (4) Conclusions: The high prevalence of HD in pedigree cats should be considered and screening pedigree cats for HD is recommended before they are used in breeding programs.

## 1. Introduction

Hip dysplasia (HD) is a very well described condition in dogs, especially among certain breeds [1,2,3,4]. However, HD is much less described in cats, most likely because cats can often much better compensate for pelvic limb lameness and hide their pain and, as a result, owners are less likely to notice this condition. The reported prevalence of HD varies, among different studies, from 6.6% [5] to 32% [6], as well as among breeds, with the incidence being higher in purebred cats (12.3%) [5] than in Domestic Shorthair cats (5.8%) [6]. One study showed that Maine Coon (MCO) cats were most likely to be affected with up to 21% prevalence of HD [5]. Recently, a large study of 2708 MCO cats in the Orthopedic Foundation for Animals (OFA) registry showed a prevalence of HD in MCO of 24.9%, slightly higher in males (27.3%) than females (23.3%) [7]. In this study, bilateral HD was more severe than unilateral and overall prevalence increased with age [7]. A recent study using 20 years of data from a radiographic health screening program of 5038 pedigree registered MCO cats showed a prevalence of 37.4%, with no sex predilection, but the severity of HD increased with age and body mass [8]. Other larger body type breeds, such as Persian cats, have also been reported to be more likely affected due to their large body type [5]. Studies showing the prevalence among larger breeds, such as Norwegian Forrest (NFO) cats, Siberian (SIB) cats and British Shorthair cats, are missing.

The clinical signs associated with HD in cats are usually gradual in onset, mild and easily missed by owners [9]. The main clinical signs are decreased activity and reluctance to jump on higher places, reluctance to use stairs and to squat when defecating and defecation outside of the litter box; signs of pelvic limb lameness are rare. Performing a thorough feline orthopedic exam can be challenging and attention should be paid to, for example, resentment to handling and showing signs of aggression when being touched [10,11].

The diagnosis of HD in cats is mainly based on the physical exam, including the assessment of coxofemoral laxity (Ortolani sign) and hip radiographs [7,12]. The evaluation of feline pelvic radiographs for the presence of HD differs from those in dogs. Cats have physiologically shallower acetabulum and the degenerative changes of the femoral head and neck are less marked and develop later than in dogs [12].

Patellar luxation has been reported in cats less frequently than in dogs, where Devon Rex, Siamese, British Shorthair and Abyssinian cats are overrepresented [13,14,15,16]. It has been reported as both a congenital condition and a result of trauma [13,17]. Both unilateral and bilateral patellar luxation have been reported and are most commonly occurring medially [13,15,17]. Concurrent HD and patellar luxation have also been reported in cats, with a weak association between these conditions [17,18,19,20].

The clinical signs of patellar luxation in cats are similar to those of HD. Additionally, the animal may display an abnormal gait with external rotation of the stifle or occasional locking of the joint [12,17]. Patellar luxation in cats is often diagnosed based on the palpation of the stifle joints [21].

A lumbosacral transitional vertebra (LTV) is a congenital spinal anomaly (abnormal vertebral segment possessing both lumbar and sacral morphological characteristics), located between the last lumbar vertebra and the first sacral vertebra with normal morphology [22,23,24,25]. The prevalence of LTV in a population of 405 cats, investigated in the absence of spinal disease, was 5.9% [26]. In dogs, LTV has been reported to be a risk factor for the development of degenerative lumbosacral stenosis and with possible biomechanics alteration of the lumbosacral junction, accelerating degenerative changes [22,23,27]. There are three types of LTV, lumbar (type 1), intermediate (type 2) and sacral (type 3). In symmetric LTV, both transverse processes are of the same type, while, in an asymmetric LTV, they are not; however, the classification of LTV types is inconsistent in veterinary literature. A link has been described between asymmetrical LTV and severe hip dysplasia in dogs [24] and a similar tendency has been found in cats [25]. However, the frequency of hip dysplasia in both dogs and cats with LTV was very similar to those without [25,28]. A recent study showed that LTV in cats could be considered a risk factor for developing lumbosacral stenosis [26].

The main objective of this study was to find the prevalence of feline HD, patellar luxation and LTV in pedigree cats in the Czech Republic. It might be useful when deciding if pedigree cats should be screened before their use in breeding programs as it is commonly done in Scandinavia (PawPeds https://pawpeds.com/healthprogrammes/hd.html (accessed on 17 August 2021)) [29] and North America (OFA, https://www.ofa.org/diseases/hip-dysplasia#screeningprocedures (accessed on 17 August 2021)) [30].

## 2. Materials and Methods

**Figure 1:**
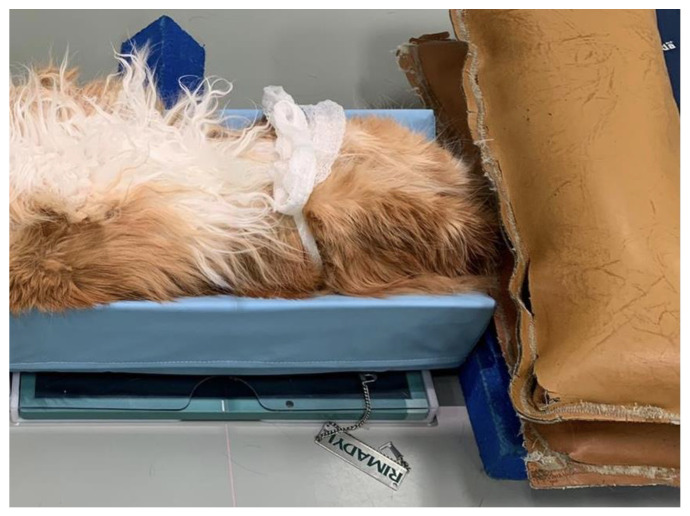
The cats were placed in dorsal recumbency in a V-trough with sternum aligned and the pelvis straight for obtaining ventrodorsal extended leg radiographs.

Cats were recruited prospectively at the Small Animal Clinic at the University of Veterinary and Pharmaceutical Sciences Brno, CZ, from April 2019 to July 2020. All cats had to be pedigree cats and at least 10 months old to be recruited for the study. Cats were excluded from the study if they were pregnant, nursing, had a history of trauma or any contraindications for sedation. Sedation was obtained using 0.3 mg/kg of butorphanol (Butomidor, Richter Pharma, Wels, Austria) and 8 mcg/kg of dexmedetomidine (Dexdomitor, Zoetis, Charles City, IA, USA) and/or 2 mg/kg of alfaxalone (Alfaxan, Jurox, Rutherford, Australia) intramuscularly (IM) as needed. In all cats sedated with dexmedetomidine, reversal was achieved using atipamezole (Antisedan, Zoetis, USA) IM at half the total volume. All sedated cats had blink and swallowing reflexes, as well as regular respiration and did not require intubation. All cats had three-view radiographs of the hips: ventrodorsal in both extension and flexion and a laterolateral projection. For a lateral image of the pelvis, the cats were positioned on the table with the right side down for a right lateral image. For ventrodorsal extended leg radiographs, the cats were placed in dorsal recumbency in a V-trough to keep the vertebral column and sternum aligned and the pelvis in a straight position with legs extended (Figure 1). For ventrodorsal flexed leg radiographs, V-trough was used to help keep the patient’s vertebral column and sternum aligned with legs in flexed position.

All cats had their hips evaluated for an Ortolani sign to assess the coxofemoral laxity and both stifles for the presence of patellar luxation. The examination and palpation were performed prior to sedation by the same person (PP) throughout the study. The former was considered either negative or positive and the latter was graded based on the severity of luxation. Radiographs were taken using the radiography system Proteus XR/a (GE, Boston, MA, USA) and computed radiography Capsula XL (Fuji, Tokyo, Japan). All radiographs were made using an 18 × 24 cm^2^ cassette with a resolution of 1770 × 2370 pixels and saved in the PACS system in DICOM format.

**Figure 2:**
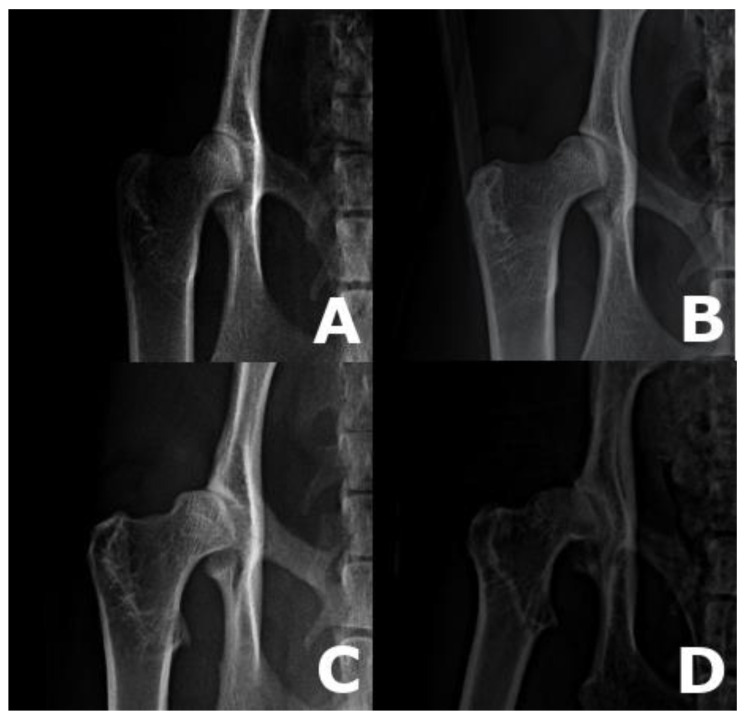
Examples of hip radiographs. (**A**) Grade 0—normal hip with no evidence of feline HD. (**B**) Grade 1—hip with acetabulum covering < 50% of the femoral head. (**C**) Grade 2—moderate radiographic signs associated with feline HD, including shallow acetabulum and deformation of the femoral head, with some evidence of new bone formation around the joint. (**D**) Grade 3—severe radiographic signs associated with feline HD with very poor joint congruency, deformation of the femoral head and major changes associated with new bone formation around the joint.

All radiographs were evaluated at the end of the study by the Department of Diagnostic Imaging at the Small Animal Clinic at the University of Veterinary and Pharmaceutical Sciences Brno, CZ in JiveX DICOM Viewer (VISUS Technology Transfer, GmbH, Bochum, Germany). Evaluation of the hip joint radiographs was performed according to the published grading protocol by Paw Peds (https://pawpeds.com/healthprogrammes/hd.html (accessed on 17 August 2021)) [29] and individual hip joints were assessed based on the degree of subluxation and osteoarthritic changes into four grades (Figure 2) as described by Low et al., 2019 [8].

**Figure 3:**
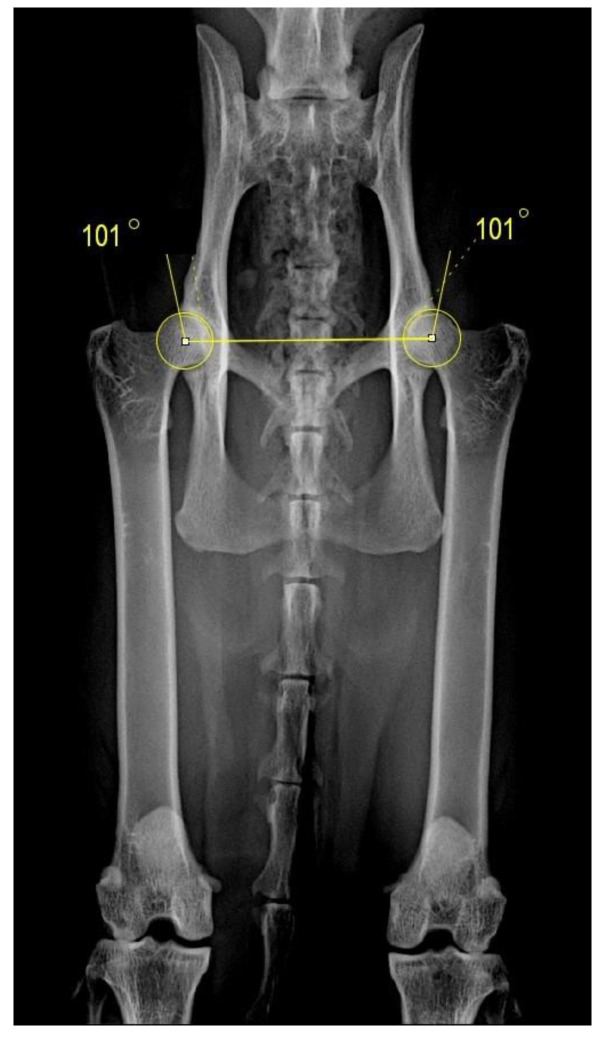
Measurement of the Norberg angle in the extended view.

The Norberg angle (NA) was also assessed in a template with circles of varying diameter superimposed on the radiographs to determine the center of each femoral head with a line being traced through the centers of the femoral heads. The center of each femoral head was connected to the cranial–dorsal rim of the acetabulum. The NA was calculated at the medial side by the intersection of the two lines in both extended (Figure 3) and flexed view [6]. Originally, the measurement of NA was developed for VD flexion in dogs and later adopted for VD extension view [31].

All cats had their patella examined by manual palpation prior to sedation and the degree of patellar luxation was graded on a scale from 0 to 4 based on the criteria, as described by Loughin et al., 2006 [21], and LTV was characterized based on the appearance of the transverse process and divided into three groups, as described by Fluckiger et al., 2006 [23].

All pedigree cat owners had to fill in a questionnaire on the day of the examination with the following questions:

1. Has your cat had any mobility problems, e.g., with jumping or climbing in the past 6 months?
2. Have you noticed any decreased or other changes in activity in your cat in the past 6 months?
3. Have you noticed any increased vocalization when you touched your cat in the past 6 months?
4. Has your cat had any problems or changes in defecation in the past 6 months?

Statistical analysis of the NA was performed using the paired *t*-test and *p*-values ≤ 0.05 were considered significant.

## 3. Results

A total of 107 pedigree cats were prospectively enrolled in this study (71 females and 36 males). There were 85 MCO, 13 NFO, 5 Burmilla cats (BML), 3 SIB and 1 Oriental Shorthair cat (OSH). The mean age of the pedigree cats was 35 months (SD ± 22.4). The mean bodyweight was 5.59 kg (SD ± 1.47).

The prevalence of HD in all pedigree breeds was 46.7% (50/107 cats), with 39.3% (42/107) being in the right hip joint and 37.4% (40/107) in the left hip joint (Table 1). In 36.4% (39/107) of cats with HD, the dysplasia was bilateral. In most cases, the hip dysplasia was classified as grade 1 (17.8% (19/107) in the right hip joint and 18.7% (20/107) in the left hip joint), or grade 2 (16.8% (18/107) in the right hip joint and 11.2% (12/107) in the left hip joint). However, 4.7% (5/107) of the right hip joints and 7.5% (8/107) of the left hip joints showed signs of grade 3 HD. Bodyweight did not influence the prevalence of feline HD (*p* = 0.28).

The mean NA in the extended view of pedigree cats was 96.87 (±3.45) in grade 0, 87.82 (±3.24) in grade 1, 80.20 (±6.02) in grade 2 and 74.20 (±8.50) in grade 3 (Table 2). The mean NA in the flexed view of pedigree cats was 97.99 (±3.36) in grade 0, 91.38 (±3.72) in grade 1, 87.06 (±6.23) in grade 2 and 84.64 (±5.85) in grade 3. There was a significant difference between the NA measurement in extended and flexed views (*p* < 0.0001). The mean number of radiographs that were needed to be taken for good diagnostic quality was 2.8 (SD ± 1.1) in extended view and 1.3 (SD ± 0.5) in flexed view.

Medial patellar luxation was noted in 32.7% (35/107) of the pedigree cats and was bilateral in 29.9% of the cases (32/107). In most cases, patellar luxation was only grade 1 (26.2% (28/107) in the right patella and 25.2% (27/107) in the left patella) and grade 2 (5.6% (6/107) in the right patella and 4.7% (5/107) in the left patella). Grade 3 luxation was only observed in the right patella in 0.9% (1/107) of cases. The contralateral limb was not affected and no patients were classified as grade 4 (Table 3). Nineteen pedigree cats were affected by both feline HD and patellar luxation (17.8%).

**Figure 4:**
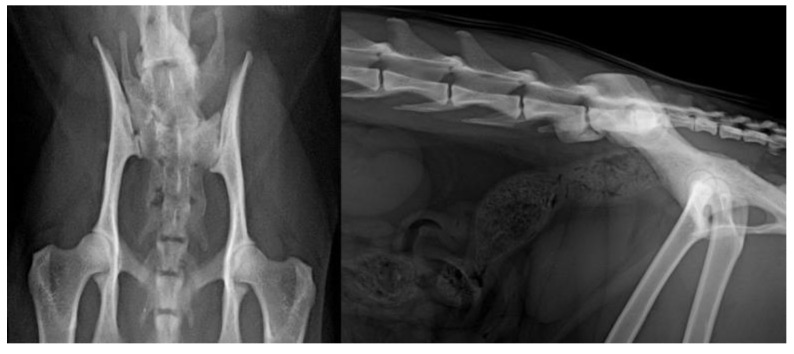
The presence of an asymmetrical type III LTV on ventrodorsal and laterolateral projections.

The presence of LTV was noted in 7.5% (8/107) of the pedigree cats, four in MCO, three in NFO and one in BML. Out of the eight LTVs, four were type II and four were type III (Figure 4).

Only in 1/107 cats, the breeder noticed an increased vocalization when touching their cat. No other changes in activity, defecation or movement were noticed.

## 4. Discussion

The reported prevalence of feline HD varies from 6.6% [5] to 32% [6] among different studies. There is also a large difference between breeds, with MCO being the most affected with a reported prevalence of 21–37.4% [5,7,8]. The prevalence of hip joint dysplasia was 46.7% in our study, which is much higher than previously reported. The majority was ≤ grade 2; however, 4.7% of the right hip joints and 7.5% of the left hip joints were classified as grade 3. Bilateral HD was seen in 78% (39/50) of cats with HD. The higher prevalence of HD in our study could be influenced by our breed bias, as most of the cats in this study were MCO cats, that seem to have higher prevalence of HD, based on previous reports.

The mean number of radiographs that were needed to be taken for good diagnostic quality was 2.8 (SD ± 1.1) in extended view and 1.3 (SD ± 0.5) in flexed view. In the VD flexion “frog leg position”, the femoral head is forced deeply into the acetabulum and, in cat with hip joint laxity, can lead to diminished NA. Standard positioning is required for HD grading in cats. The ventrodorsal flexion view is an alternative position suitable especially for old animals in which severe coxofemoral degenerative joint disease is expected or where anesthesia is not allowed. It can mask hip joint laxity, but it is excellent for the early detection of osteoarthrosis on femoral head and neck [31,32]. There was a significant difference between the NA angle in the extended and flexed views (*p* < 0.0001). These findings suggest that there is a need for extended views for hip radiographs and that cats require heavier sedation to accomplish good diagnostic quality radiographs to assess HD. It also highlights the importance to mention which view was used to obtain pelvis radiographs.

Hip dysplasia was reported in three DSH littermates, showing that genetics likely plays a role [33]. HD has been recognized as an inherited disease with a polygenic mode of inheritance [5]. Therefore, screening of HD in pedigree cats before their use in breeding programs should be considered. Moreover, the effect of environment and nutrition on the development of HD in cats requires further studies.

Interestingly, despite the high prevalence of HD and patellar luxation in these cats, no changes in movement, activity and defecation habits were noted by the breeders in 6 months prior to the study. Only in 1/107 cats, the breeder noticed an increased vocalization when touching their cat. The reasons could be that cats are much better at hiding their pain, their owners cannot notice the clinical signs, or both. This suggestion is supportive of a previous study, suggesting that the clinical signs associated with HD in cats are usually gradual in onset, mild and easily missed by the owners [9].

Patellar luxation was noted in 32.7% of the pedigree cats and was present bilaterally in 91.4% of the cats. It was classified as grade 1 or 2 in most patients. Nineteen pedigree cats were affected by both feline HD and patellar luxation (17.8%), which is similar to previously reported results [20].

The presence of LTV was noted in 7.5% (8/107) of the cats. It has been previously reported that the frequency of hip dysplasia in cats with LTV was very similar to those without [25]. The prevalence of LTV in our study was low and the frequency of LTV and HD was therefore not looked at.

## 5. Conclusions

The prevalence of HD in all pedigree cats was 46.7%, of which 78% of cats had bilateral dysplasia. The dysplasia was mainly from mild (grade 1) to moderate (grade 2); however, 6.1% of hip joints showed signs of severe hip dysplasia (grade 3) in MCO and SIB cats. Patellar luxation was noted in 32.7% of the pedigree cats, was present bilaterally in 91.4% and was grade 1 or 2 in most cats. The presence of LTV was noted in 7.5% of the pedigree cats. The high prevalence of HD in pedigree cats should be considered and screening pedigree cats for HD is recommended before they are used in breeding programs.

## Figures and Tables

**Table 1:** Prevalence of hip joint dysplasia in different pedigree breeds and total prevalence in pedigree cats.

Breed	Right Hip Joint				Left Hip Joint			
Breed	Grade 0	Grade 1	Grade 2	Grade 3	Grade 0	Grade 1	Grade 2	Grade 3
BML	2	2	1	0	2	2	1	0
MCO	50	15	16	4	52	17	9	7
NFO	11	2	0	0	11	0	2	0
OSH	1	0	0	0	1	0	0	0
SIB	1	0	1	1	1	1	0	1
**Total**	**65**	**19**	**18**	**5**	**67**	**20**	**12**	**8**
**%**	**60.7**	**17.8**	**16.8**	**4.7**	**62.6**	**18.7**	**11.2**	**7.5**

BML, Burmilla cats; MCO, Maine Coon; NFO, Norwegian Forrest; OSH, Oriental shorthair; SIB, Siberian cats.

**Table 2:** Prevalence of hip joint dysplasia in different pedigree breeds and total prevalence in pedigree cats.

Norberg Angle	Grade 0	Grade 1	Grade 2	Grade 3
VD extended view	96.87 (±3.45)	87.82 (±3.24)	80.20 (±6.02)	74.20 (±8.50)
VD flexed view	97.99 (±3.36)	91.38 (±3.72)	87.06 (±6.23)	84.64 (±5.85)

**Table 3:** Prevalence of patellar luxation in different pedigree breeds and total prevalence in pedigree cats.

Breed	Right Patella					Left Patella				
	Gr. 0	Gr. 1	Gr. 2	Gr. 3	Gr. 4	Gr. 0	Gr. 1	Gr. 2	Gr. 3	Gr. 4
BML	2	1	2	0	0	2	2	1	0	0
MCO	59	21	4	1	0	60	21	4	0	0
NFO	9	4	0	0	0	11	2	0	0	0
OSH	1	0	0	0	0	1	0	0	0	0
SIB	1	2	0	0	0	1	2	0	0	0
**Total**	**72**	**28**	**6**	**1**	**0**	**75**	**27**	**5**	**0**	**0**
**%**	**67.3**	**26.2**	**5.6**	**0.9**	**0.0**	**70.1**	**25.2**	**4.7**	**0.0**	**0.0**

BML, Burmilla cats; MCO, Maine Coon; NFO, Norwegian Forrest; OSH, Oriental shorthair; SIB, Siberian cats. Gr., grade.

## Data Availability

All data can be found in PACS at the Department of Diagnostic Imaging at the University of Veterinary Sciences Brno.
